# IGF2BP2 regulates gastric cancer radiotherapy resistance through HIF1α-mediated glycolysis

**DOI:** 10.3389/fonc.2025.1512177

**Published:** 2025-05-16

**Authors:** Qi Zhang, Xiaoyu Liu, Yukan Chen, Ruilin Wang, Shuangyan Zhao, Yuting Tian, Shuping Li, Xiaojun Liu

**Affiliations:** ^1^ The First Clinical Medical College of Gansu University of Chinese Medicine, Lanzhou, Gansu, China; ^2^ Xi'an International Medical Center Hospital, Xian, Shanxi, China; ^3^ Department of Radiation Oncology II, Gansu Provincial Hospital, Lanzhou, Gansu, China; ^4^ The Third Clinical Medical College of Lanzhou University, Lanzhou, Gansu, China; ^5^ The First Clinical Medical College of Lanzhou University, Lanzhou, Gansu, China

**Keywords:** IGF2BP2, HIF1α, glycolysis, radioresistance, gastric cancer

## Abstract

This study reveals the core mechanism by which insulin-like growth factor 2 mRNA-binding protein 2 (IGF2BP2) drives tumor progression and radiotherapy resistance in gastric cancer (GC) through m^6^A-dependent regulation of hypoxia-inducible factor 1α (HIF1α). Clinical analyses show that IGF2BP2 is significantly overexpressed in gastric cancer tissues, and its expression level is closely associated with tumor size and poor patient prognosis. Functional studies demonstrate that elevated expression of IGF2BP2 accelerates the transition of gastric cancer cells from the G1 phase to the G2/M phase of the cell cycle and markedly enhances cell proliferation and migration through anti-apoptotic effects. Mechanistically, IGF2BP2 specifically binds to the m^6^A-modified “GGACU” motif within the coding region of HIF1α mRNA, positively regulating HIF1α mRNA stability and protein expression in an m^6^A-dependent manner. High IGF2BP2 expression significantly enhances glycolytic activity in gastric cancer cells, while overexpression of HIF1α partially rescues the suppression of malignant phenotypes caused by IGF2BP2 knockdown, indicating that HIF1α serves as a key downstream effector mediating the oncogenic role of IGF2BP2 in gastric cancer. Furthermore, knockdown of IGF2BP2 significantly increases radiosensitivity by exacerbating DNA damage and enhancing oxidative stress.

## Introduction

m^6^A is the most common RNA epigenetic modification, which can affect RNA folding, stability, degradation, etc., thereby participating in splicing, translation, apoptosis, and other processes (1). Recent studies have found that m^6^A-modified regulators are involved in a variety of biological processes (e.g., cell differentiation, tissue development), especially in the development and progression of a variety of malignant tumors (e.g., gastric and breast cancers), and their modulators have shown promising therapeutic targets in human cancers (2, 3). Insulin-like growth factor 2 mRNA-binding protein 2 (IGF2BP2), a newly discovered third class of m^6^A-reading proteins, is mainly localized in the cytoplasm and has a unique KH structural domain and flanking structure that recognizes and binds m^6^A modifications in a novel way (4). IGF2BP2, as an RNA-binding protein, stabilizes its target mRNAs by targeting and recognizing the m^6^A modifications. We screened the main target genes of IGF2BP2 for AKT, MYC, and hypoxia-inducible factor 1α (HIF1α) through the RM2Target database (5) and found that all these genes are highly involved in glycolysis. Glycolysis is not only associated with many hallmark features of cancer such as proliferation, angiogenesis, and immune escape but is also closely related to late tumor progression, treatment resistance, and poor prognosis (6). Therefore, studies on the regulation of glycolysis activity by IGF2BP2 are important in elucidating radiotherapy resistance.

Gastric cancer is the fifth most common cancer and the fourth most common cause of death worldwide (7). Radiotherapy plays an important role in the comprehensive treatment of gastric cancer, but radiotherapy resistance is an important factor leading to tumor recurrence and metastasis and affecting the efficacy of radiotherapy (8, 9). The main causes of radiotherapy resistance include hypoxia in the tumor microenvironment, the antioxidant system in cancer cells, and the upregulation of DNA repair proteins (10). Oxygen acts as a natural radiosensitizer, and oxygenated cells are approximately three times more radiosensitive than hypoxic cells (11). However, hypoxia occurs in 90% of solid tumors and has been defined as one of the most important reasons for radiotherapy failure since 1953 (12, 13). In a hypoxic environment, cancer cells obtain energy by inhibiting aerobic respiration and promoting glycolysis, and the levels of glycolysis-related proteins are strongly correlated with resistance to radiotherapy (14, 15). Glycolysis also enhances radioresistance by promoting DNA damage repair, which provides a theoretical basis for the study of glycolytic inhibitory factors to increase the sensitivity to radiotherapy (16). Enhanced glycolysis in solid tumors significantly increases the secretion of lactic acid, which has antioxidant capacity, and its concentration is positively correlated with radiotherapy resistance (17). Therefore, inhibition of aerobic glycolysis may be a means to promote sensitization to cancer radiotherapy.

Reactive oxygen species (ROS) is a general term for reactive oxygen-containing compounds (ROCs), which originate from the oxidative respiration of the mitochondria, including superoxide anion, hydrogen peroxide, and free radicals, and is an indispensable mediator of indirect DNA damage caused by photon radiation (18). The level of ROS is tightly regulated by the cellular antioxidant defense system. When ROS levels are too high, the antioxidant system protects biomolecules such as DNA, proteins, and membrane lipids of tumor cells from oxidative damage by neutralizing ROS, thus maintaining the normal function of tumor cells (19). Activation of mitochondrial pyruvate dehydrogenase (PDH) enhances the level of oxidative phosphorylation and increases ROS production, which facilitates the effect of radiotherapy (20). Pyruvate dehydrogenase kinases (PDKs), on the other hand, inhibit PDH activity and exert the opposite effect. Inhibition of PDK activity does increase the sensitivity of gastric cancer cells to radiotherapy (21). Hypoxia-inducible factor 1 (HIF1) is recognized as a major regulator of the hypoxia response, which promotes cellular adaptation to hypoxia through transcriptional regulation of its target genes and can also promote key aspects of tumor progression, including angiogenesis, metabolism, and metastasis (22). It has been shown that HIF1α can indirectly inhibit PDH through transcriptional activation of PDK, inhibit the tricarboxylic acid cycle and oxidative phosphorylation, and contribute to the shift of cellular glucose metabolism from oxidative phosphorylation to glycolysis, which is not conducive to the effect of radiotherapy (23, 24). Therefore, the study of the regulation of HIF1α pathway activity is of great significance in elucidating radiotherapy resistance.

In this study, we found that the m^6^A-reading protein IGF2BP2 increases the stability and translation of HIF1α mRNA transcripts by targeting the recognition of m^6^A, which in turn promotes the activity of enzymes related to glucose metabolism, inhibits mitochondrial oxidative phosphorylation, reduces the generation of ROS, and ultimately promotes glucose metabolism and radiotherapy resistance in gastric cancer. Taking gastric cancer as the research object, we linked epigenetics, glucose metabolism, and radiotherapy resistance, aiming to reveal the new mechanism of radiotherapy resistance in gastric cancer and explore new ways to improve the efficacy of radiotherapy in gastric cancer.

## Materials and methods

### Immunohistochemical staining and scoring analysis

Gastric cancer prognostic tissue microarrays (ZL-STC1601) were purchased from Vio Biotechnology Company (Shanghai, China). The immunohistochemical (IHC) staining kit (Bios Biological Technology Company, China) and anti-IGF2BP2 antibody (Wuhan Sanying Biotechnology Co., Ltd., Wuhan, China) were used to stain the microarrays. The number of positives and the intensity of staining in each section were converted into corresponding values by histochemistry score to achieve semiquantitative staining of tissues. The H-score is the data between 0 and 300 (a higher score indicates the integrated positive intensity). Statistical analysis and survival curves were plotted using GraphPad Prism 9.5 software. All clinicopathologic data are displayed in **Table 1**.

**Table 1:** GC patients’ clinicopathological characteristics with IGF2BP2 expression.

Characteristics	Clusters	Number of cases	IGF2BP2		*p*-value
		80	Low	High	
Age	<55	34	19	15	0.366
	≥55	46	21	25	
Gender	Male	53	26	27	0.813
	Female	27	14	13	
Tumor diameter	<5	50	30	20	0.021*
	≥5	30	10	20	
TNM stage	I/II	39	20	19	0.823
	III/IV	41	20	21	
Lymph metastasis	Positive	50	25	25	0.952
	Negative	30	15	15	
Histological grade	Low/middle	38	18	20	0.654
	High	42	22	20	
Survival time	<5	45	17	28	0.013*
	≥5	35	23	12	

P<0.05.

### Cell lines and cell culture

MKN74 and HGC-27 were purchased from Procell Wuhan, and MKN45, AGS cells, and Gastric Epithelial Cell Line 1 (GES1) were provided free of charge by the Laboratory of Gansu Provincial People’s Hospital. All cell lines were identified by short tandem repeat (STR) analysis and detected for *Mycoplasma* contamination. Cells were cultured in RPMI 1640 medium supplemented with 10% fetal bovine serum (FBS) and 1% penicillin/streptomycin (P/S) and placed in an incubator at 37°C and 5% CO_2_. Cell culture reagents were purchased from Biological Industries, Israel.

### Lentiviral construction and cell transfection

Short hairpin RNA (shRNA) targeting human IGF2BP2 was ligated into the hU6-MCS-CMV-puro (cloning site: *Age*I, *Eco*RI) vector to silence IGF2BP2 expression. Overexpression of IGF2BP2 was constructed using the Ubi-MCS-3FLAG-CBh (cloning site: *Bam*HI, *Age*I) vector. The lentivirus was synthesized by Jikai Biotechnology (Shanghai, China). MKN74 and HGC-27 cells were incubated with viral particles 10^7^ TU/mL overnight. The samples were then incubated with 2–3 μg/mL of puromycin for 2 weeks to establish stable transfectors. After confirmation of silencing, overexpression efficiency by real-time fluorescence quantitative PCR (RT-qPCR), and protein blotting analysis, the cells were used for the subsequent experiments. shRNA sequences are listed in **Supplementary File 1**: **Supplementary Table S1**.

### CCK8 cell proliferation assay

Dimethyloxalylglycine (DMOG, MedChemExpress, USA) is a small molecule that stabilizes HIF1α expression under normoxic conditions, and it achieves this by inhibiting HIF-degrading enzymes, thereby preventing HIF1α degradation. MKN74 gastric cancer cells were seeded at approximately 8,000 cells per well in 96-well plates and cultured in a complete medium under standard incubator conditions. After 24 h of incubation, the waste liquid of each well was aspirated, and DMOG was diluted in RPMI 1640 medium to final concentrations of 0, 50, 100, 200, 400, and 800 μM. Each concentration group included six replicate wells. At 24 h, 10 μL of CCK8 reagent was added to each well under light-protected conditions. The 96-well plates were placed in an incubator at 37°C, and the absorbance values were measured using an enzyme marker after 1 h of incubation. The IC50 values were calculated and plotted using GraphPad Prism 9.5 software.

### Real-time fluorescence quantitative PCR assay

Total RNA was extracted from cells using the M5 Universal RNA Mini Kit (Mei5bio, Beijing, China). Reverse transcription was performed using the M5 Sprint qPCR RT Kit. Real-time qPCR was conducted on a LightCycler Nano System using M5 HiPer SYBR Green Premix EsTaq (Mei5bio, Beijing, China). GAPDH was used as the internal control gene. The relative RNA expression levels were calculated using the 2^−ΔΔCt^ method. The relevant primer sequences are provided in **Supplementary File 1**: **Supplementary Table S2**.

### Western blotting

The control and transfected MKN74 and HGC-27 cells were harvested and lysed using enhanced RIPA lysis buffer containing protease inhibitors (Mei5bio, Beijing, China). Total protein concentration was measured using a BCA protein assay kit (Boster, Wuhan, China). Equal amounts of protein were separated by SDS-PAGE using SWE rapid high-resolution electrophoresis buffer (Sevier, Wuhan, China) and transferred onto PVDF membranes (Merck Millipore, Ireland). After blocking with skimmed milk, the membranes were incubated with primary and secondary antibodies to detect the expression of target proteins. Antibodies against GAPDH and β-actin were used as loading controls. Additional reagents and antibodies are listed in **Supplementary File 1**: **Supplementary Table S3**.

### Cell proliferation assay

The control and transfected MKN74 and HGC-27 cells were seeded into six-well plates at a density of approximately 1 × 10^5^ cells per well. A 2× EdU working solution was prepared according to the instructions of the EdU assay kit (Beyotime, Shanghai, China), mixed with an equal volume of culture medium, and added to the wells. The cells were incubated for 2 h for EdU labeling. After labeling, the culture medium was removed, and the cells were fixed with 1 mL of 4% paraformaldehyde (Beyotime, Shanghai, China) for 15 min at room temperature. The stained cells were observed under a fluorescence microscope (Olympus BX51, Japan) to assess cell proliferation. ImageJ software (NIH, USA) was used to calculate the percentage of EdU-positive cells and the average fluorescence intensity.

### Migration assay

The control and transfected MKN74 and HGC-27 cells were seeded in a serum-free medium into Falcon Transwell chambers with 8-μm pore size (Corning Life Sciences, Acton, MA, USA) at a density of approximately 2 × 10^4^ cells per well. A total of 700 μL of medium containing 10% fetal bovine serum (FBS) was added to the lower chamber, and 200 μL of serum-free medium containing the cells was added to the upper chamber. The chambers were then incubated at 37°C with 5% CO_2_ for 24 h. After incubation, non-migrated cells on the upper surface of the membrane were carefully removed using a cotton swab. The membranes were fixed with 4% paraformaldehyde for 30 min, followed by staining with 0.1% crystal violet for 15 min. Excess dye was washed off, and migrated cells were photographed under a microscope. Cell migration levels were quantified using ImageJ software.

### Flow cytometry assay

The control and transfected MKN74 and HGC-27 cells were seeded into six-well plates at approximately 2 × 10^5^ cells per well and cultured for 24 h. After incubation, cells from each group were stained at room temperature in the dark using the Cell Cycle and Apoptosis Analysis Kit (Elabscience, Wuhan, China). The stained cells were then analyzed using a FACSCalibur flow cytometer. Dead cells and the relative proportions of early and late apoptotic cells were analyzed using FlowJo 10.8.1 software. The distribution of cells in different cell cycle phases was analyzed using the ModFit 5.0 software.

### RNA pull-down assay

To verify the binding between IGF2BP2 and HIF1α, we used an RNA Pull-Down Kit (FitGene, Guangzhou, China) for the assay. Biotin-labeled RNA probes corresponding to HIF1α mRNA (full-length, CDS-WT, and CDS-MUT) were synthesized using the Biotin Labeling Mix Kit (Roche Biologicals, Basel, Switzerland). The biotinylated probes were incubated with MKN74 cell lysates, followed by the addition of pretreated streptavidin-coated magnetic beads to capture the RNA–protein complexes. Non-specifically bound proteins were removed by multiple washing steps, and the captured complexes were subsequently analyzed by Western blotting to detect the presence of IGF2BP2.

### Glucose and lactate testing

The control and transfected MKN74 and HGC-27 cells were seeded into 24-well plates at a density of 2 × 10^3^ cells per well and cultured in the complete medium for 24 h. After 24 h, the cells were irradiated with a dose of 4 Gy using a 6-MV medical electron linear accelerator and then incubated for another 24 h. Subsequently, the cells were processed according to the instructions of the Glucose and Lactic Acid Content Detection Kit (Solarbio, Beijing, China). The treated samples were transferred to 96-well plates, and absorbance at 530 nm was measured using a microplate reader. Data were analyzed and visualized using GraphPad Prism 9.5 software.

### Clone formation experiment

To evaluate the radiosensitivity of gastric cancer cells, MKN74 and HGC-27 cells were seeded into six-well plates 48 h after transfection at densities of 100, 200, 400, 800, or 1,600 cells per well. The cells were cultured in the complete medium in an incubator at 37°C. After 24 h of incubation, the wells seeded with 200, 400, 800, and 1,600 cells were irradiated with doses of 2, 4, 6, and 8 Gy, respectively, using a 6-MV medical electron linear accelerator. Following irradiation, the medium was discarded and replaced with fresh complete medium. The medium was changed every 2–3 days, and cell growth was monitored until visible colonies formed. Colonies were then fixed, stained with crystal violet, and washed. Colonies containing more than 50 cells were counted, and the results were analyzed using ImageJ software to calculate radiobiological parameters.

### ROS detection

The control and transfected MKN74 and HGC-27 cells were seeded into six-well plates at a density of 1 × 10^4^ cells per well and cultured in the complete medium. After 24 h of incubation, the medium was replaced with the fresh complete medium, and the cells were irradiated with a 6-MV medical electron linear accelerator at a dose of 4 Gy. Following an additional 48 h of incubation, DCFH-DA was diluted 1:1,000 in RPMI 1640 medium to prepare a working solution with a final concentration of 10 μmol/L. The medium was removed, and approximately 1.5 mL of the diluted DCFH-DA solution was added to each well. Cells were incubated at 37°C for 20–30 min. After incubation, the cells were washed three times with RPMI 1640 medium to remove any residual DCFH-DA that had not entered the cells. Finally, the cells were observed under an inverted fluorescence microscope and photographed for documentation.

### Statistical analysis

All data are expressed as mean ± standard deviation (SD). Statistical analyses were performed using SPSS 25.0 (SPSS, USA) and GraphPad Prism 9.5 (GraphPad, USA). Pearson correlation analysis was performed to assess the relationship between IGF2BP2 and HIF1α expression levels. Kaplan–Meier curve analysis was used to assess overall survival, and group differences were evaluated using *t*-tests, one-way ANOVA, or chi-square tests, as appropriate. *P <*0.05 was considered statistically significant. Statistical significance was denoted as follows: ns, not significant (*P* > 0.05); *, *P* < 0.05; **, *P* < 0.01; ***, *P* < 0.001; ****, *P* < 0.0001.

## Results

### Upregulation of IGF2BP2 is associated with low survival in gastric cancer patients

**Figure 1:**
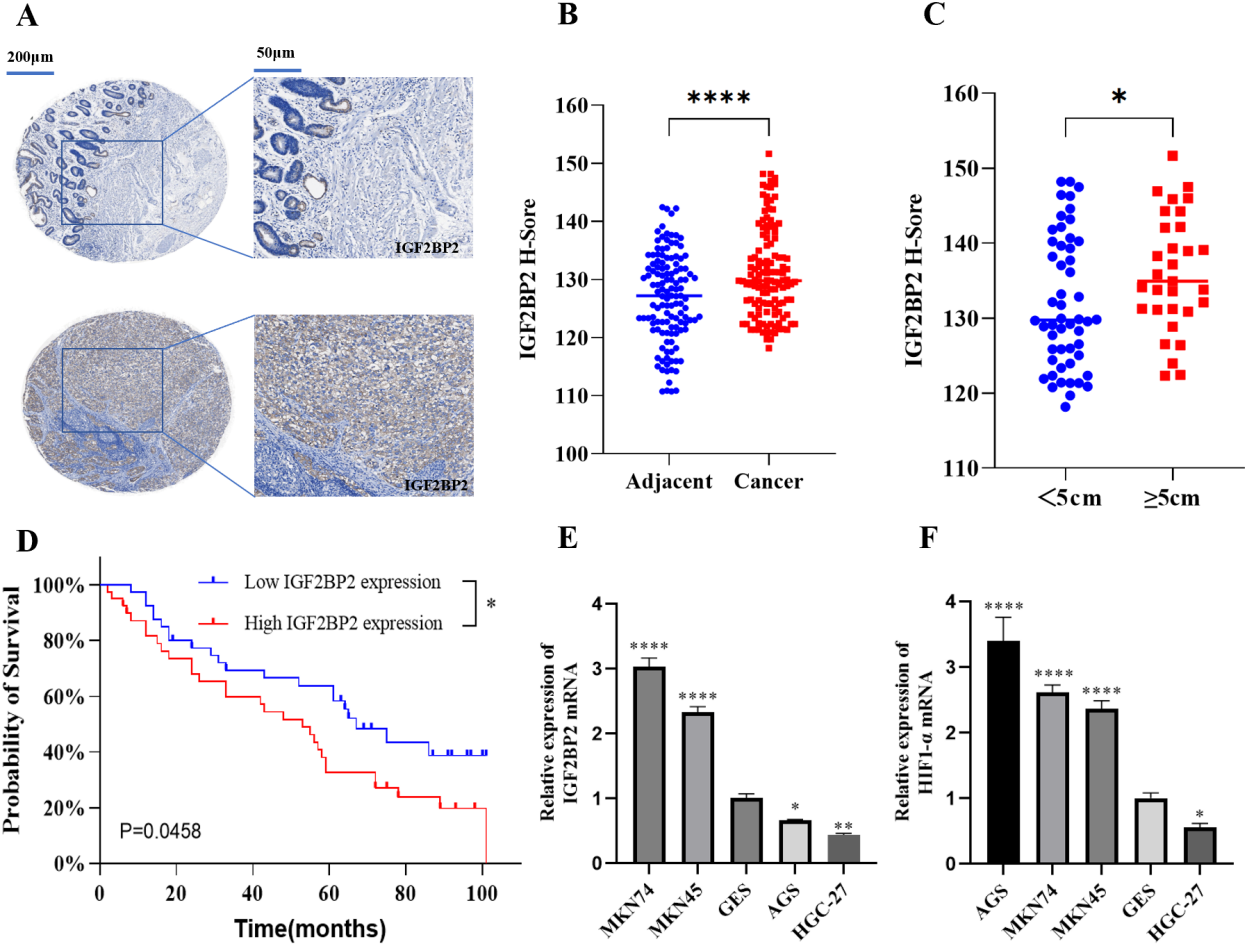
Expression and clinical significance of IGF2BP2 in gastric cancer tissues and cells. **(A)** IHC staining of IGF2BP2 in gastric cancer tissue microarrays compared to adjacent tissues. **(B)** IHC staining intensity in cancer and adjacent tissues was quantified using H-scores; higher H-scores indicate stronger overall positive staining. **(C)** Correlation between IGF2BP2 expression and tumor diameter in gastric cancer. **(D)** Overall survival of gastric cancer patients with high or low IGF2BP2 expression analyzed using the Kaplan-Meier method and log-rank test. **(E, F)** Expression of IGF2BP2 and HIF1α in various gastric cancer cell lines detected by RT-qPCR. *P<0.05, **P<0.01, ****P<0.0001.

To investigate the expression of IGF2BP2, we performed IHC analysis on 80 paired gastric cancer and adjacent non-tumor tissue specimens. IHC staining revealed that IGF2BP2 was significantly overexpressed in gastric cancer tissues (**Figure 1A**), with high expression observed in 71.25% (57 out of 80) of tumor samples compared to the matched adjacent tissues (**Figure 1B**). Subsequently, we examined the correlation between IGF2BP2 expression and clinicopathologic characteristics of the patients. IGF2BP2 expression was significantly higher in patients with tumor diameters larger than 5 cm, whereas it was lower in those with tumors smaller than 5 cm (**Figure 1C**). Moreover, high IGF2BP2 expression was associated with poorer overall survival in gastric cancer patients (**Figure 1D**). However, no significant association was found between IGF2BP2 expression and other clinical parameters, including age (<55 vs. ≥55 years), gender, lymph node metastasis (negative vs. positive), tumor stage (I–IV), or histologic grade (low, intermediate, high). RT-qPCR analysis showed that IGF2BP2 and HIF1α mRNA levels were significantly upregulated in MKN74 and MKN45 cell lines compared to normal gastric epithelial cells (GES1) (**Figure 1E**), but were significantly downregulated in HGC-27 cell lines (**Figure 1F**). Based on these findings, MKN74 and HGC-27 cell lines were selected for the subsequent experiments.

### IGF2BP2 promotes gastric cancer cell proliferation and migration

**Figure 2:**
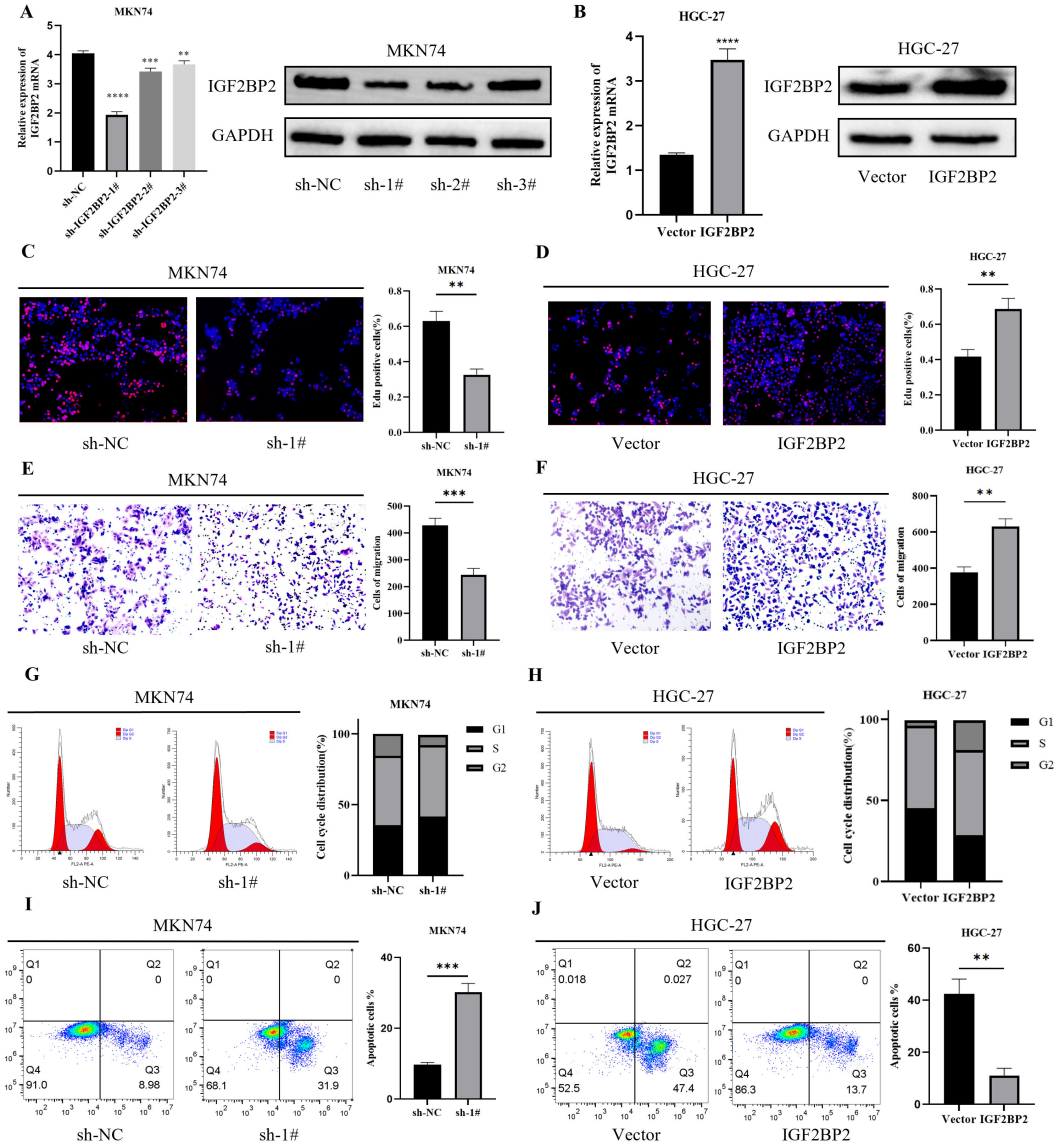
IGF2BP2 affects the malignant behavior of gastric cancer cells. **(A, B)** IGF2BP2 expression in MKN74 and HGC-27 cells after IGF2BP2 transfection was detected by RT-qPCR and Western blotting. **(C, D)** Cell proliferation assay using EdU immunofluorescence staining. **(E, F)** Cell migration analyzed using the Transwell assay. **(G, H)** Cell cycle distribution (G1, S, and G2 phases) analyzed by FACS after IGF2BP2 transfection. **(I, J)** Apoptosis analysis of gastric cancer cells after IGF2BP2 transfection, as detected by FACS. **P<0.01, ***P<0.001, ****P<0.0001.

To explore the role of IGF2BP2 in the malignant progression of gastric cancer (GC) cells, we used MKN74 cells for IGF2BP2 silencing and HGC-27 cells for IGF2BP2 overexpression. Transfection efficiency was confirmed by RT-qPCR and Western blotting. The results showed that IGF2BP2 expression was significantly downregulated in MKN74 cells (**Figure 2A**) and upregulated in HGC-27 cells (**Figure 2B**). The EdU assay demonstrated that IGF2BP2 silencing inhibited GC cell proliferation (**Figure 2C**), whereas its overexpression promoted proliferation (**Figure 2D**). Transwell migration assays revealed that IGF2BP2 silencing reduced the migration of MKN74 cells (**Figure 2E**), while overexpression enhanced the migration of HGC-27 cells (**Figure 2F**). These findings strongly support that high IGF2BP2 expression promotes the malignant phenotype of GC cells. We further analyzed the effects of IGF2BP2 on cell cycle progression and apoptosis using flow cytometry. IGF2BP2 silencing increased the proportion of MKN74 cells in the G1 phase compared to negative controls (**Figure 2G**), whereas its overexpression in HGC-27 cells promoted the transition from the G1 phase to the G2 phase (**Figure 2H**). Additionally, IGF2BP2 silencing enhanced apoptosis in MKN74 cells (**Figure 2I**), while its overexpression reduced apoptosis in HGC-27 cells (**Figure 2J**). These results further elucidate the mechanism by which IGF2BP2 regulates the malignant behavior of GC cells.

### IGF2BP2 stabilizes HIF1α mRNA by recognizing m^6^A mechanism in GC cells

**Figure 3:**
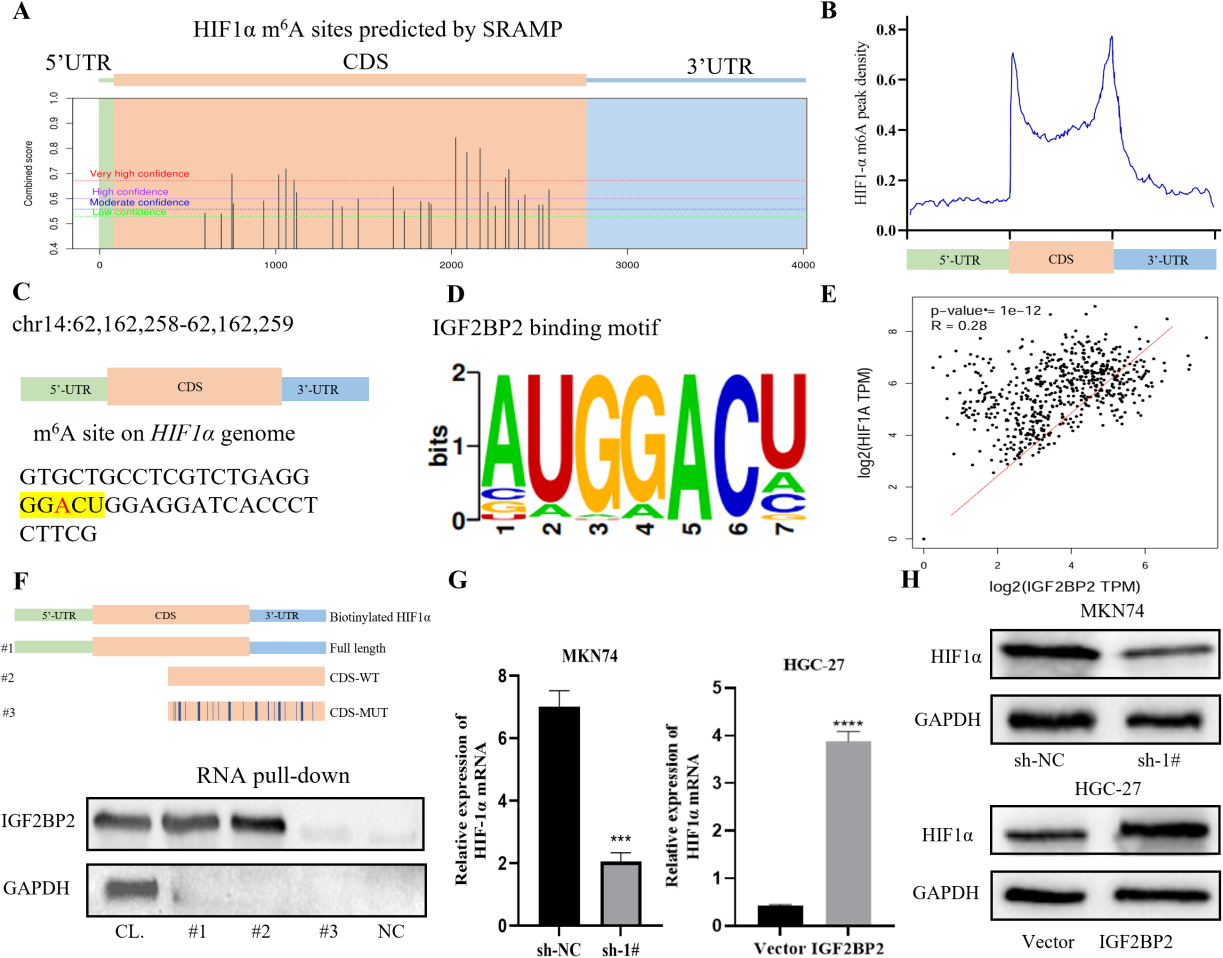
HIF1α is a target of IGF2BP2. **(A)** An online prediction tool (SRAMP, http://www.cuilab.cn/sramp) identified m6A modification sites in the HIF1α gene. **(B)** Metagenomic mapping showed the distribution of m6A peaks in HIF1α mRNA. **(C)** In the CDS region of the HIF1α gene, the m6A modification site was identified as ‘GGACU’ (chr14: 62,162,258–62,162,259). **(D)** The binding motif of IGF2BP2 was consistently identified as the ‘GGACU’ sequence by RMBase v2.0 (https://rna.sysu.edu.cn/rmbase/). **(E)** Clinical correlation analysis using GEPIA (http://gepia.cancer-pku.cn/index.html). **(F)** In MKN74 cells, RNA pull-down assays were performed using cell lysates (CL), full-length biotinylated HIF1α (1#), HIF1α with or without a mutation at the m6A site in the CDS region (2#, 3#), and beads only as a negative control (NC). IGF2BP2 was detected by Western blot. **(G, H)** HIF1α expression in MKN74 and HGC-27 cells transfected with IGF2BP2 was measured by RT-qPCR and Western blot. ***P<0.001, ****P<0.0001.

Previous studies have identified HIF1α as a well-established factor contributing to radiotherapy resistance. In this study, we used the online prediction tool SRAMP (http://www.cuilab.cn/sramp) (25) to identify several potential m^6^A modification sites on the HIF1α gene (**Figure 3A**). The distribution of m^6^A peaks on HIF1α is shown as a metagenomic map in **Figure 3B**, which demonstrates that, across all conditions, there was significant enrichment of m^6^A peaks in the coding sequence (CDS) and 3′ untranslated region (3′ UTR). Within the CDS region of the HIF1α gene, we identified a conserved m^6^A modification motif “GGACU” at chr14:62,162,258–62,162,259 (**Figure 3C**). According to RMBase v2.0 (https://rna.sysu.edu.cn/rmbase) (26), the IGF2BP2 binding motif also corresponds to the “GGACU” sequence (**Figure 3D**). Clinical correlation analysis using GEPIA (http://gepia.cancer-pku.cn/index.html) (27) revealed a positive correlation between IGF2BP2 and HIF1α expression in gastric adenocarcinoma (STAD) samples (**Figure 3E**). RNA pull-down assay demonstrated that IGF2BP2 was significantly enriched in MKN74 gastric cancer cells and predominantly bound to the CDS region of HIF1α mRNA. This binding was significantly reduced when the m^6^A site was mutated (**Figure 3F**). Finally, RT-qPCR and Western blotting showed that silencing of IGF2BP2 decreased HIF1α expression, whereas overexpression of IGF2BP2 increased HIF1α expression (**Figures 3G, H**). Collectively, these findings indicate that IGF2BP2 regulates HIF1α mRNA expression in an m^6^A-dependent manner.

### IGF2BP2 recovery assay with overexpression of HIF1α

**Figure 4:**
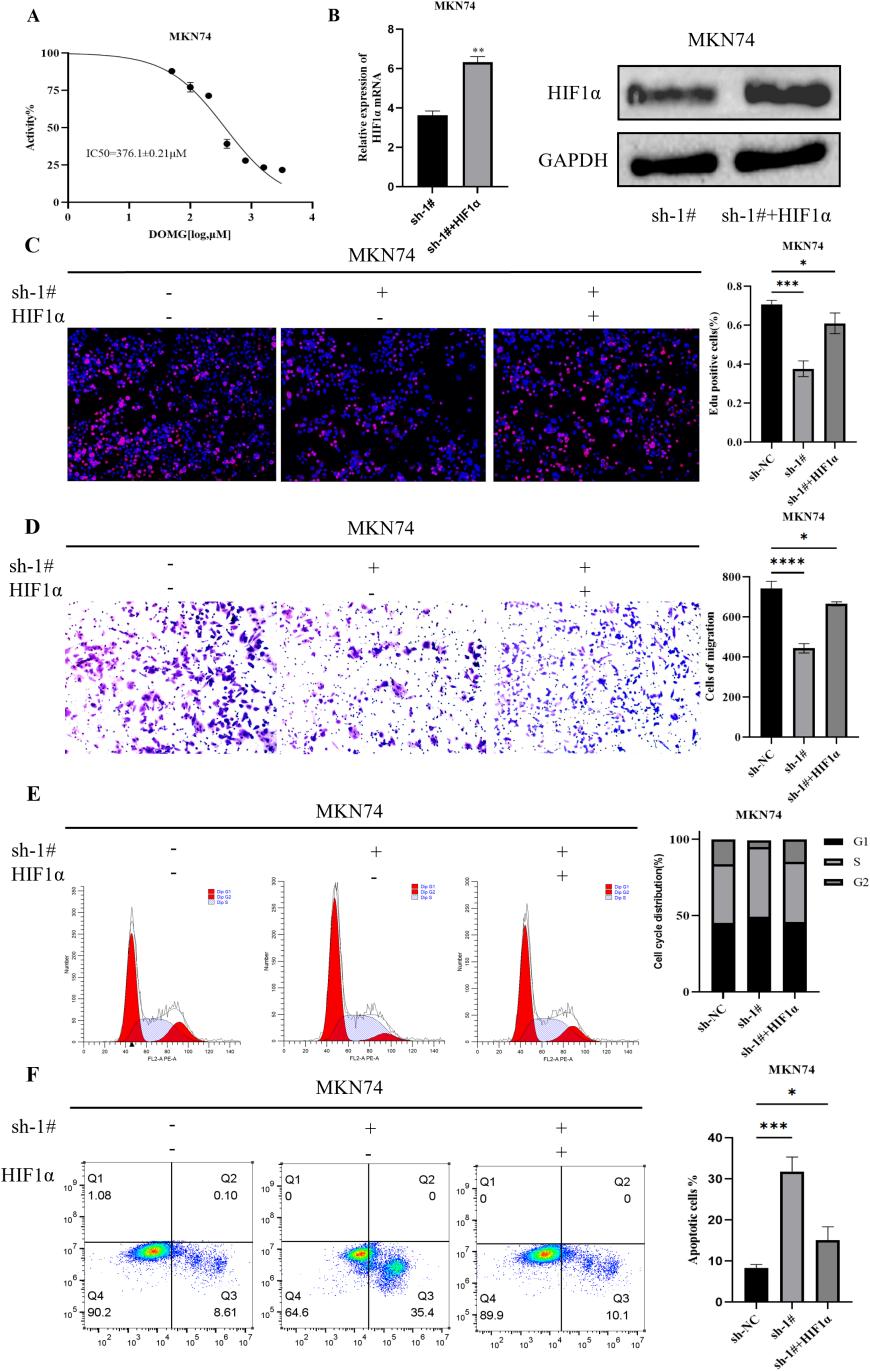
IGF2BP2 regulates gastric cancer progression through HIF1α. **(A)** The IC50 value of the HIF1α agonist DMOG was determined in IGF2BP2-knockdown MKN74 cells. **(B)** HIF1α expression in DMOG-treated MKN74 cells was evaluated by RT-qPCR and Western blot analysis. **(C)** Cell proliferation was assessed using EdU immunofluorescence staining. **(D)** Cell migration was evaluated using the Transwell assay. **(E)** The distribution of gastric cancer cells in the G1, S, and G2 phases after co-transfection with IGF2BP2 and overexpressed HIF1α was analyzed by FACS. **(F)** The apoptosis rate of gastric cancer cells after co-transfection with IGF2BP2 and overexpressed HIF1α was analyzed by FACS. *P<0.05, **P<0.01, ***P<0.001, ****P<0.0001.

To investigate whether HIF1α contributes to IGF2BP2-induced GC cell growth, we performed a rescue assay. MKN74 cells transfected with IGF2BP2 were first treated with varying concentrations of the HIF1α agonist DMOG (0 to 800 µM). After 24 h, absorbance was measured and a dose–response curve was generated (**Figure 4A**). The IC50 value of DMOG in IGF2BP2-knockdown MKN74 cells was determined to be 376.1 ± 0.21 µM. Next, RT-qPCR and Western blot (WB) analyses confirmed that DMOG treatment upregulated HIF1α expression in MKN74 cells transfected with IGF2BP2 (**Figure 4B**). Subsequently, cellular phenotype rescue assays were conducted. EdU assay results indicated that overexpression of HIF1α restored the proliferative capacity of MKN74 cells with stable IGF2BP2 knockdown (**Figure 4C**). Similarly, Transwell assays showed that inhibition of cell migration caused by IGF2BP2 silencing was partially reversed by HIF1α overexpression (**Figure 4D**). The effects of HIF1α overexpression on cell cycle progression and apoptosis were further examined by flow cytometry. The results demonstrated that HIF1α overexpression attenuated the apoptosis rate in IGF2BP2-silenced MKN74 cells (**Figure 4E**). Moreover, overexpression of HIF1α restored the cell cycle transition from the G1 to the G2 phase compared to the respective negative controls (**Figure 4F**). Together, these findings suggest that IGF2BP2 promotes GC cell growth, at least in part, through HIF1α.

### IGF2BP2 affects HIF1α-mediated glycolysis

**Figure 5:**
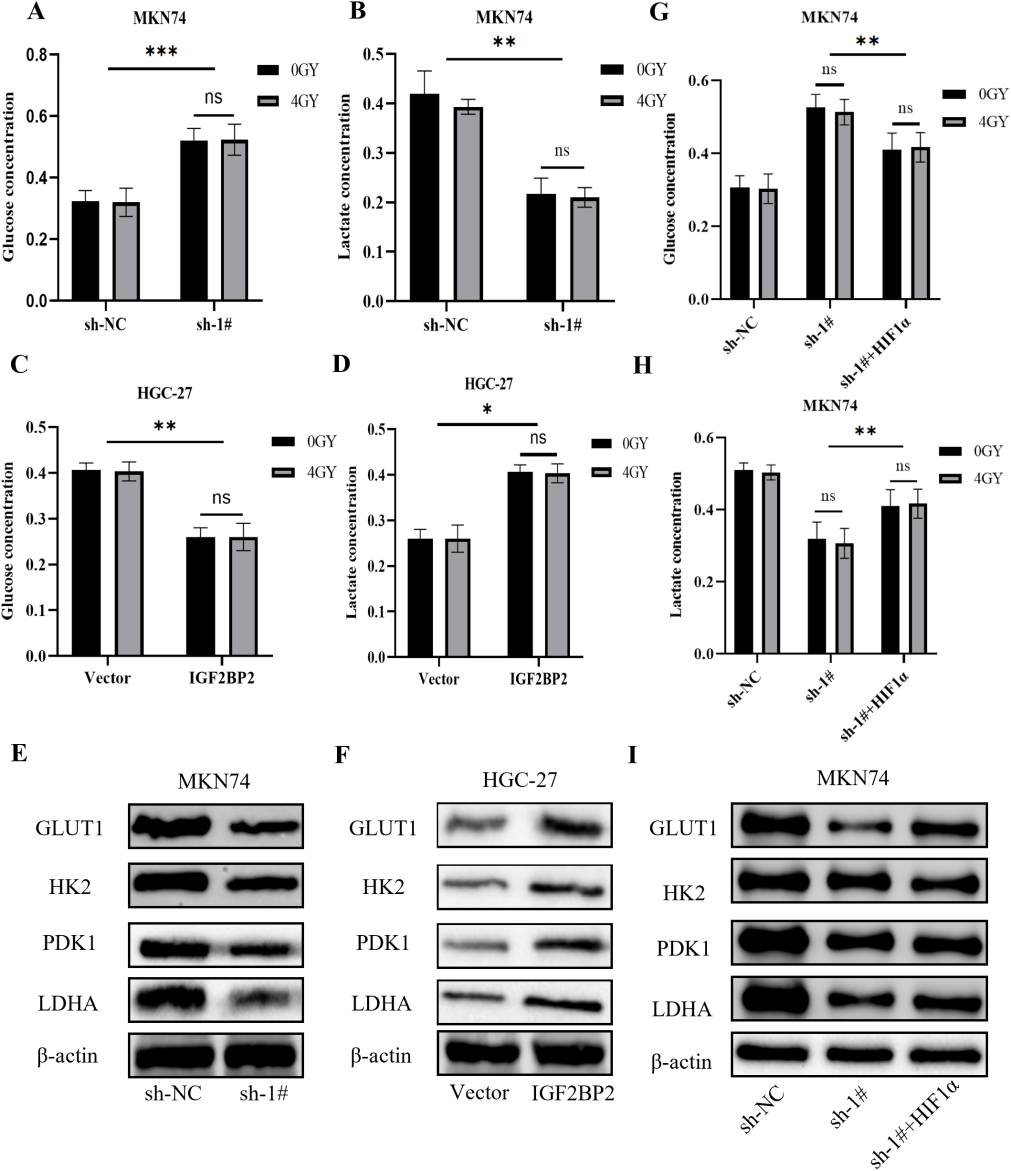
Effects of IGF2BP2 on glucose metabolism in gastric cancer cells. **(A-D)** Glucose consumption and lactate production were measured in gastric cancer MKN74 and HGC-27 cells before and after IGF2BP2 transfection, with or without radiation treatment. **(E, F)** Western blot analysis was performed to assess the protein expression levels of GLUT1, HK2, PDK1, and LDHA in MKN74 and HGC-27 cells transfected with IGF2BP2. **(G, H)** Glucose consumption and lactate production were measured in MKN74 cells transfected with IGF2BP2, treated with DMOG, and subjected to irradiation. **(I)** Western blot analysis was performed to evaluate the expression levels of GLUT1, HK2, PDK1, and LDHA in MKN74 cells co-treated with IGF2BP2 and DMOG. *P<0.05, **P<0.01, ***P<0.001.

It has been shown that HIF1α can indirectly inhibit PDH through the transcriptional activation of PDK, thereby promoting a metabolic shift from the tricarboxylic acid (TCA) cycle and oxidative phosphorylation to glycolysis. This shift leads to reduced production of ROS, which may negatively impact the efficacy of radiotherapy. Our group has long been interested in exploring whether metabolic alterations in gastric cancer contribute to radiotherapy resistance. Therefore, we investigated glycolytic changes during the interaction between IGF2BP2 and HIF1α. Silencing IGF2BP2 resulted in reduced glucose consumption and lactate production in MKN74 cells (**Figures 5A, B**), whereas overexpression of IGF2BP2 enhanced glucose consumption and lactate production in HGC-27 cells (**Figures 5C, D**). However, upon combining these treatments with 4 Gy of radiation, we observed no significant changes in glucose consumption or lactate production, which was consistent with our prior expectations (*P* = ns). Western blot analysis revealed that silencing IGF2BP2 led to decreased expression of downstream glycolytic genes regulated by HIF1α, including Glucose Transporter 1 (GLUT1), Hexokinase 2 (HK2), Pyruvate Dehydrogenase Kinase 1 (PDK1), and Lactate Dehydrogenase A (LDHA), which corresponded with reduced HIF1α levels (**Figure 5E**). Conversely, overexpression of IGF2BP2 upregulated these genes in parallel with increased HIF1α expression (**Figure 5F**). **Figures 5G–I** demonstrate that glycolytic impairment caused by IGF2BP2 knockdown was partially rescued by HIF1α overexpression in MKN74 cells, even under radiation treatment, indicating that radiation did not impact this regulatory process. Collectively, these findings further confirm the regulatory role of the IGF2BP2–HIF1α–glycolysis axis in gastric cancer metabolism and suggest that this mechanism is not affected by radiation exposure.

### Effect of IGF2BP2 on colony formation in irradiated gastric cancer cells

**Figure 6:**
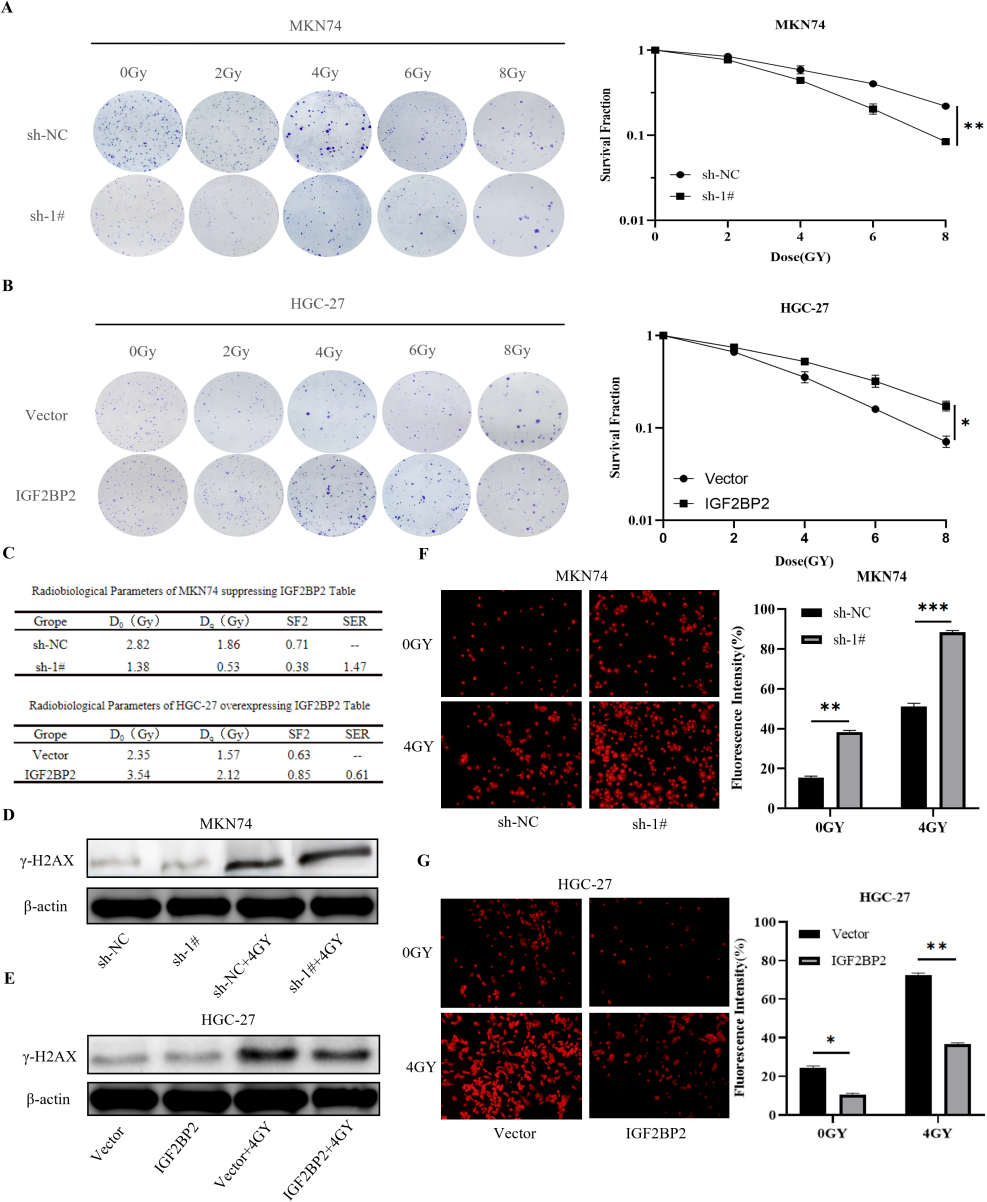
Effect of IGF2BP2 on irradiated gastric cancer cells. **(A, B)** Clonogenic survival assays of transfected MKN74 and HGC-27 cells exposed to different doses of radiation. **(C)** Summary of radiobiological parameters calculated from the survival curves of MKN74 and HGC-27 cells. **(D, E)** γ-H2AX expression was evaluated by Western blot in transfected MKN74 and HGC-27 cells treated with 4 Gy irradiation. **(F, G)** Detection of ROS generation in transfected MKN74 and HGC-27 cells co-treated with 4 Gy irradiation. *P<0.05, **P<0.01, ***P<0.001.

To evaluate whether IGF2BP2 influences the radiosensitizing effect in gastric cancer cells, clonogenic assays were performed using established stable cell lines subjected to radiation treatment. The data were analyzed using the multitarget single-hit model: SF = 1 − (1 − exp(−D/D_0_))*ⁿ*, where Dq = D_0_ × ln(*N*). Cell survival curves were generated using GraphPad Prism 9.5 software to calculate the mean lethal dose (D_0_), the shoulder region of the survival curve (Dq), and the sensitizing enhancement ratio (SER). The survival fraction at 2 Gy (SF2) is a commonly used indicator of cellular radiosensitivity (28), with values ranging from 0 to 1, with lower SF2 values indicating greater radiosensitivity. We fitted survival curves (**Figures 6A, B**) using the multitarget single-hit model based on the clonogenic assay data and generated a table of radiobiological parameters (**Figure 6C**). The results showed that the clonogenic survival of cells in the radiation + IGF2BP2 knockdown group was significantly reduced, and their radiosensitivity was significantly increased compared to the radiation-only group. In HGC-27 cells, compared to the radiation-only group, cells in the radiation + IGF2BP2 overexpression group exhibited significantly enhanced clonogenic ability and reduced radiosensitivity. These findings indicate that silencing IGF2BP2 enhances the radiosensitivity of gastric cancer cells, with an SER of 1.47, demonstrating a clear radiosensitizing effect.

### Effect of IGF2BP2 on γ-H2AX production in irradiated gastric cancer cells

DNA double-strand breaks (DSBs) represent a major form of cellular damage induced by radiation and trigger the formation of γ-H2AX, a well-established marker of DSBs (29). In this study, the expression levels of γ-H2AX protein were assessed by immunoblotting in IGF2BP2-modified gastric cancer cell lines before and after exposure to radiation (4 Gy). In MKN74 cells, a significant increase in γ-H2AX expression was observed following radiation combined with IGF2BP2 silencing, compared to radiation alone (**Figure 6D**). In contrast, in HGC-27 cells, γ-H2AX expression significantly decreased when IGF2BP2 was overexpressed in combination with radiation, compared to radiation alone (**Figure 6E**). These findings indicate that silencing IGF2BP2 enhances radiation-induced DNA damage, as evidenced by increased γ-H2AX levels, thereby suggesting a potential radiosensitizing effect of IGF2BP2 knockdown.

### Effect of IGF2BP2 on ROS generation in irradiated gastric cancer cells

The primary mechanism of radiotherapy involves the generation of ROS, and tumor cells exposed to hypoxic conditions often develop a ROS-resistant phenotype (30). The hypoxic microenvironment in solid tumors allows cancer cells to evade radiation-induced cytotoxicity and contributes to the development of radioresistance. These clonally derived, resistant cells may serve as a source of tumor recurrence. To investigate this, we treated MKN74 gastric cancer cells with a combination of IGF2BP2 silencing and radiation and measured intracellular ROS levels using fluorescence microscopy. The results showed that ROS production was significantly increased in the radiation plus IGF2BP2-silenced group compared to the radiation-only group (**Figure 6F**). In contrast, in HGC-27 cells, ROS generation was significantly reduced in the radiation plus IGF2BP2-overexpression group compared to radiation alone (**Figure 6G**). These findings suggest that the combined intervention of radiation and IGF2BP2 silencing enhances ROS production in gastric cancer cells, thereby improving the effectiveness of radiotherapy.

## Discussion

Radiotherapy sensitivity in gastric cancer is influenced by numerous factors. Factors such as gene expression, epigenetic alterations, and substance metabolism are all associated with radiotherapy sensitivity (31). Methylated RNA nucleotides widely exist in all kingdoms of life, which play crucial roles in pathophysiological processes (32). The effect of epigenetic alterations on cellular radiotherapy sensitivity involves a variety of epigenetic regulatory factors and mechanisms, and these studies help us to better understand the regulatory mechanisms of radiation sensitivity and provide a theoretical basis for individualized radiation therapy. For example, the study of Visvanathan et al. (33) found that silencing the methyltransferase METTL3 reduced DSB repair and increased the sensitivity of glioma stem cells (GSCs) to γ radiation. In cervical cancer, FTO-mediated demethylation regulates the expression of β-catenin and promotes the activity of Excision Repair Cross-Complementation Group 1 (ERCCI), which makes cervical squamous carcinoma resistant to chemoradiotherapy (34). The antitumor effect of radiation is largely dependent on the oxygen effect. When killing tumors, on the one hand, it directly damages the cellular DNA, and on the other hand, it stimulates the ROS, which indirectly exerts a cell-killing effect on the cells. The HIF signaling pathway is closely associated with tumor cell glucose metabolism and radiosensitivity (35). As a recognized radiotherapy resistance factor, it can promote the shift of cellular glucose metabolism from oxidative phosphorylation to glycolysis, which is not conducive to the effect of radiotherapy. In pancreatic ductal adenocarcinoma, targeting the lactate and HIF1α signaling pathways from tumor cells attenuates radioresistance (36). Our study supports these findings by demonstrating that regulation of HIF1α expression and lactate production in gastric cancer by the methylated reading protein IGF2BP2 exerts a radiosensitizing effect. Therefore, the study of HIF pathway activity regulation is of great significance in elucidating the resistance of gastric cancer to radiotherapy. The regulation of HIF pathway activity is complex and multifactorial. More and more studies have shown that overexpression of HIF1α exists in cancer cells even under normoxic conditions (37). Our study also confirmed the normoxic expression of HIF1α in various cell lines of gastric cancer. An in-depth study of other regulatory factors of HIF1α besides normoxia has important radiotherapy scientific significance and clinical translational value.

Our bioinformatics prediction revealed that m^6^A modification of HIF1α mRNA is located in the “GGACU” sequence on chromosome 14 and is also likely to be a downstream target gene of IGF2BP2. Our RNA pull-down experiments confirmed that IGF2BP2 mainly binds to the CDS region of HIF1α mRNA, and its binding ability is significantly weakened when the m^6^A site is mutated. This further corroborates that IGF2BP2 relies on m^6^A modification to regulate the stabilization and translational expression of HIF1α mRNA. m^6^A can be involved in cancer-related signaling pathways by regulating the expression of important molecules, affecting cell proliferation, metastasis, and metabolism, among other processes (38, 39). In glioma tissues and cells, SUMOization of IGF2BP2 promotes HIF1α expression, which in turn leads to cancer cell invasion and angiogenesis (40). In pancreatic cancer, IGF2BP2 promotes cancer cell proliferation and metastasis through activation of the PI3K–Akt signaling pathway (41). In colorectal cancer, knockdown of IGF2BP2 attenuates MYC-mediated glycolysis in colorectal cancer cells, and the LINRIS–IGF2BP2–MYC axis is a promising therapeutic target for colorectal cancer (42). Our study also confirmed the abnormally high expression of IGF2BP2 in pathological tissues of gastric cancer, which further promotes tumor cell proliferation, migration, and radioresistance by affecting the cell cycle, apoptosis, and glycolysis. Weng et al. demonstrated that small-molecule inhibitors targeting the IGF2BP family [e.g., Chemical Wnt Inhibitor 1-2 (CWI1-2)] could competitively bind to KH domains to disrupt m^6^A-dependent functions, showing remarkable efficacy in acute myeloid leukemia models (3). Li et al. demonstrated that the functional roles of m^6^A modifications in metabolic diseases, aging, infections, and cancer represent only the tip of the iceberg. Further identification or development of novel therapies targeting m^6^A modifications requires high-throughput screening of small-molecule compound libraries or chemical synthesis approaches (43). Luo et al. proposed in non-small cell lung cancer studies that combining HIF1α inhibitors with programmed cell death protein 1 (PD-1) antibodies enhances T-cell infiltration, suggesting potential dual antitumor effects of targeting HIF1α (44). Together with our current findings, developing compounds that specifically inhibit IGF2BP2–HIF1α interaction may emerge as a novel therapeutic strategy for gastric cancer. Furthermore, these discoveries not only provide new theoretical insights into the functional role of the IGF2BP2–HIF1α signaling axis in gastric carcinogenesis and progression but also establish an experimental foundation for exploring potential therapeutic targets against this pathway.

Focusing on the frontiers and scientific issues, this study is dedicated to elucidating the molecular mechanism of HIF1α overexpression in gastric cancer cells under normoxic conditions, expanding the new understanding of m^6^A modification in the regulation of glucose metabolism and radiotherapy resistance in gastric cancer, and revealing the effect of a new class of m^6^A reading proteins on the sensitivity of gastric cancer to radiotherapy. IGF2BP2 may be a molecular indicator for predicting radiosensitization and even the efficacy of radiotherapy in gastric cancer. Our study helps to deepen our understanding of cancer radiotherapy resistance, provides a new theoretical basis for the study of radiosensitization therapy for gastric cancer, and lays the foundation for the design of IGF2BP2-specific inhibitors for future intervention in gastric cancer.

Undeniably, our study still has some limitations. We plan to establish patient-derived xenograft (PDX) models using immunodeficient mice to better recapitulate the heterogeneity of human cancers and the tumor microenvironment. In these models, we will systematically evaluate the impact of IGF2BP2 expression on tumor progression and glycolytic activity. Small-molecule inhibitors targeting IGF2BP2 will be combined with radiotherapy to assess their potential in enhancing radiosensitivity in mice. These investigations are ongoing and will be pursued in future research.

## Conclusion

**Figure 7:**
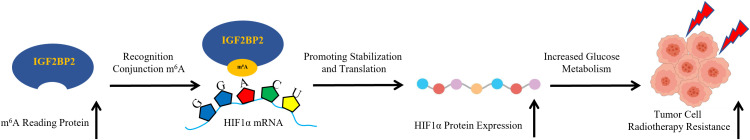
Schematic illustration of the IGF2BP2-HIF1α-glycolysis axis in regulating radiotherapy resistance in gastric cancer.

Elevated expression of IGF2BP2 promotes the stabilization and translation of HIF1α mRNA in an m^6^A-dependent manner, thereby enhancing glucose metabolism and radiotherapy resistance in gastric cancer. Our experimental findings confirm the radiosensitizing potential of targeting the IGF2BP2–HIF1α–glycolysis axis (**Figure 7**).

## Data Availability

The original contributions presented in the study are included in the article/**Supplementary Material**. Further inquiries can be directed to the corresponding authors.
