# Correction: IGF2BP2 regulates gastric cancer radiotherapy resistance through HIF1α-mediated glycolysis

**DOI:** 10.3389/fonc.2025.1669677

**Published:** 2025-09-18

**Authors:** Qi Zhang, Xiaoyu Liu, Yukan Chen, Ruilin Wang, Shuangyan Zhao, Yuting Tian, Shuping Li, Xiaojun Liu

**Affiliations:** ^1^ The First Clinical Medical College of Gansu University of Chinese Medicine, Lanzhou, Gansu, China; ^2^ Xi'an International Medical Center Hospital, Xian, Shanxi, China; ^3^ Department of Radiation Oncology II, Gansu Provincial Hospital, Lanzhou, Gansu, China; ^4^ The Third Clinical Medical College of Lanzhou University, Lanzhou, Gansu, China; ^5^ The First Clinical Medical College of Lanzhou University, Lanzhou, Gansu, China

**Keywords:** IGF2BP2, HIF1α, glycolysis, radioresistance, gastric cancer

In the published article, there was an error regarding the affiliation(s) for “Shuping Li” and “Xiaojun Liu”. As well as having affiliations “The Third Clinical Medical College of Lanzhou University, Lanzhou, Gansu, China”, they should also have “The First Clinical Medical College of Gansu University of Chinese Medicine, Lanzhou, Gansu, China;Department of Radiation Oncology II, Gansu Provincial Hospital, Lanzhou, Gansu, China”.

The original version of this article has been updated.

