# Supplementary material for: IGF2BP2 regulates gastric cancer radiotherapy resistance through HIF1α-mediated glycolysis
Source: Front Oncol. 2025 May 16;15:1512177. doi: 10.3389/fonc.2025.1512177 (PMC12134386; doi:10.3389/fonc.2025.1512177)
Supplement: Supplementary file 1 [file Table1.docx]

**Supplementary Table 1.** Sequences of shRNA

| Name | Sequence |
| --- | --- |
| sh-NC | TTCTCCGAACGTGTCACGT |
| sh-IGF2BP2#1 | AGCGCAAGATCAGGGAAATTG |
| sh-IGF2BP2#2 | AGTGAAGCTGGAAGCGCATAT |
| sh-IGF2BP2#3 | CTGAAGCATGCCGCATGATTC |

**Supplementary Table 2.** Primer sequences for qRT-PCR.

| Gene | Forward sequence (5'-3') | Reverse sequence (5'-3') |
| --- | --- | --- |
| IGF2BP2 | GCAGAAAGAGGCAGATGAGACC | ATCTCTATCTCAGCACTGGCACA |
| HIF1α | TGATTGCATCTCCATCTCCTACC | GACTCAAAGCGACAGATAACACG |
| GAPDH | GGAAGCTTGTCATCAATGGAAATC | TGATGACCCTTTTGGCTCCC |

Supplementary Table 3.List of reagents and antibodies.eagents and antibodies.

| Name | Manufacturer | Cat. no. |
| --- | --- | --- |
| IGF2BP2 | Proteintech（WuHan,China） | 11601-1-AP |
| HIF1α | Proteintech（WuHan,China） | 66730-1-Ig |
| γ-H2AX | Proteintech（WuHan,China） | 83307-2-RR |
| GAPDH | Proteintech（WuHan,China） | 60004-1-Ig |
| β-Actin | Proteintech（WuHan,China） | 81115-1-RR |
| GLUT1 | Proteintech（WuHan,China） | 21829-1-AP |
| HK2 | Proteintech（WuHan,China） | 66974-1-Ig |
| PDK1 | Proteintech（WuHan,China） | 17086-1-AP |
| LDHA | Proteintech（WuHan,China） | 21799-1-AP |
